# 2477 Targeting pulsatile load to increase exercise capacity and quality of life after TAVR for severe aortic stenosis

**DOI:** 10.1017/cts.2018.185

**Published:** 2018-11-21

**Authors:** Anupam Kumar, Julio Chirinos

## Abstract

OBJECTIVES/SPECIFIC AIMS: The objective of the study is to test the effect of oral inorganic nitrate on the primary outcomes of exercise capacity and quality of life in patients who have undergone TAVR for severe aortic stenosis. We will also test the effect of the study drug on various physiology endpoints, including systemic vasodilator response to exercise, LV diastolic function and myocardial strain, late systolic LV load and pulsatile arterial wave reflections. METHODS/STUDY POPULATION: This is a randomized double-blind crossover clinical trial, in which 24 subjects who underwent TAVR for severe AS 3 or more months before enrollment will receive the following 2 interventions, in randomized order: (1) Potassium nitrate (KNO_3_), at a dose of 12–18 mmol/day by mouth for ~4 weeks, or (2) Potassium chloride (KCl), at a dose of 12–18 mmol/day by mouth for ~4 weeks. A 1-week washout period will be introduced between the 2 interventions. RESULTS/ANTICIPATED RESULTS: We hypothesize that sustained oral administration of potassium nitrate will lead to improvement of exercise capacity and quality of life in this population.DISCUSSION/SIGNIFICANCE OF IMPACT: His study will have a significant impact on assessment and management of patients after TAVR. We will gain a better understanding of physiologic abnormalities leading to exercise intolerance after TAVR. In addition, there are currently no proven therapies that improve exercise capacity in this population.

